# Ferroptosis‐related gene signature predicts prognosis and immunotherapy in glioma

**DOI:** 10.1111/cns.13654

**Published:** 2021-05-10

**Authors:** Rong‐Jun Wan, Wang Peng, Qin‐Xuan Xia, Hong‐Hao Zhou, Xiao‐Yuan Mao

**Affiliations:** ^1^ Department of Clinical Pharmacology, Xiangya Hospital Central South University Changsha China; ^2^ Department of Respiratory Medicine, National Key Clinical Specialty, Branch of National Clinical Research Center for Respiratory Disease Xiangya Hospital, Central South University Changsha China; ^3^ Department of Pediatrics, Xiangya Hospital Central South University Changsha China; ^4^ National Clinical Research Center for Geriatric Disorders Xiangya Hospital Changsha China; ^5^ Institute of Clinical Pharmacology, Hunan Key Laboratory of Pharmacogenetics Central South University Changsha China; ^6^ Engineering Research Center of Applied Technology of Pharmacogenomics Ministry of Education Changsha China

**Keywords:** ferroptosis, gene signature, glioma, immunotherapy, prognosis, risk score

## Abstract

**Aims:**

Glioma is a highly invasive brain tumor, which makes prognosis challenging and renders patients resistant to various treatments. Induction of cell death is promising in cancer therapy. Ferroptosis, a recently discovered regulated cell death, can be induced for killing glioma cells. However, the prognostic prediction of ferroptosis‐related genes (FRGs) in glioma remains elusive.

**Methods:**

The mRNA expression profiles and gene variation and corresponding clinical data of glioma patients and NON‐TUMOR control were downloaded from public databases. Risk score based on a FRGs signature was constructed in REMBRANDT cohort and validated in other datasets including CGGA‐693, CGGA‐325, and TCGA.

**Results:**

Our results demonstrated that the majority of FRGs was differentially expressed among GBM, LGG, and NON‐TUMOR groups (96.6%). Furthermore, the glioma patients with low‐risk score exhibited a more satisfactory clinical outcome. The better prognosis was also validated in the glioma patients with low‐risk score no matter to which grade they were affiliated. Functional analysis revealed that the high‐risk score group was positively correlated with the enrichment scores for immune checkpoint blockade‐related positive signatures, indicating the critical role of glioma immunotherapy via risk score.

**Conclusion:**

A novel FRGs‐related risk score can predict prognosis and immunotherapy in glioma patients.

## Graphical abstract

A random survival forest model was constructed to obtain the risk score based on 59 ferroptosis genes in glioma patients in REMBRANDT cohort and it was validated in other datasets including CGGA‐325, CGGA‐693, and TCGA cohorts. Finally, a novel ferroptosis‐related risk score can predict prognosis and immunotherapy in glioma patients.

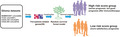

## 1 INTRODUCTION

Glioma remains the most prevalent primary malignant tumor in the central nervous system. According to the World Health Report, glioma can be classified into four grades, in which grade II and III are defined as diffuse lower‐grade gliomas (LGG), whereas grade IV glioma is also termed as glioblastoma (GBM).^1^ In general, patients with a high grade glioma often exhibit more unsatisfactory prognosis, despite discovering some prognostic biomarkers such as lncRNA FOXD1‐AS1^2^ and hemodynamic alteration.^3^ It has been demonstrated that glioblastoma possesses highly aggressive potential, with a median survival time of only 16 months,^4^ while patients with LGG have the survival range from 1 to 15 years. Despite progress in standard treatments including surgical resection, radio‐ and chemo‐therapy, glioma patients are still resistant to current available therapeutic interventions owing to highly infiltrating property for malignancy. Tumor recurrence and malignant progression are always widespread during treatment failure. Previous investigations have depicted several molecular markers, such as mutations in isocitrate dehydrogenase (IDH) and co‐deletion of the short arm of chromosome 1 and the long arm of chromosome 19 (1p/19q) are applied in molecular pathological diagnosis, treatment option, and prognostic assessment.^5^, ^6^ However, many hitherto glioma therapies targeting these molecular markers have minimal responses in clinical practice. As such, it is of urgent need to explore novel biomarkers to predict glioma prognosis.

Cell death is a critical event and participates in malignant transformation and tumor metastasis.^6^, ^7^ Ferroptosis is a novel form of regulated cell death (RCD), which was discovered by Stockwell et al^8^ in 2012. This cell death mode is very distinct from others at morphological, biochemical, and genetic levels. Generally, ferroptotic cells exhibit mitochondrial abnormality (small size and condensed membrane), iron‐dependent lethal lipid peroxide accumulation and a cassette set of altered gene expressions such as *GPX4*,^9^ *ASCL4*,^10^ *AIFM2*,^11^ and so on. There are several identified ferroptosis‐targeted reagents including ferroptosis inhibitors (eg., ferrostatin‐1, liproxstatin‐1) and ferroptosis‐inducing compounds (eg.,erastin, RSL3). Notably, it has shown that some cancer cells, which are resistant to compounds targeting traditional cell death processes, are efficiently killed by treatment with erastin and RSL3,^12^ suggesting ferroptosis induction as a promising therapeutic strategy in glioma therapy.

In addition to the typical features of ferroptosis, cells with ferroptotic stress are also accompanied by the release of proinflammatory cytokines including IFN‐γ.^13^ Actually, unlike apoptosis, it has also been demonstrated that ferroptosis seems more immunogenic due to release of damage‐associated molecular patterns (DAMPs), which in turn exacerbates inflammatory reactions.^14^ In particular, the direct evidence supporting the relationship between ferroptosis and immunity arises from the investigation that promotion of T cell‐mediated ferroptosis is able to exert potent anti‐tumor effect.^13^ It suggests that ferroptosis is involved in tumor immunotherapy. However, a comprehensive analysis of the relationship between ferroptosis and immune response in glioma is not well characterized.

Herein, our present work aimed to conduct a comprehensive evaluation of role of ferroptosis‐related genes (FRGs) signature in the prediction of prognosis and immunotherapy in glioma patients in the public databases like REMBRANDT, Chinese Glioma Genome Atlas (CGGA)‐693, CGGA‐325, and The Cancer Genome Atlas (TCGA). We drew a conclusion that the development of risk score based on FRGs has a good predictive value for survival and immunotherapy in glioma patients.

## 2 METHODS

### 2.1 Data source and processing

All the datasets with detailed clinical annotations used in our present study were obtained from Gene‐Expression Omnibus (GEO, https://www.ncbi.nlm.nih.gov/geo), The Cancer Genome Atlas (TCGA, https://portal.gdc.cancer.gov/) and Chinese Glioma Genome Atlas (CGGA, http://www.cgga.org.cn/), which were further processed via GEOquery,^15^ TCGAbiolinks,^16^ and manually, respectively. After data filtration, five eligible datasets including GSE108474‐REMBRANDT,^17^ CGGA‐693,^18^, ^19^ CGGA‐325,^20^, ^21^ TCGA‐LGG, and TCGA‐GBM with 1813 glioma patients in total were selected for further analysis. Among these datasets, GSE108474‐REMBRANDT was selected as the training set while CGGA‐693, CGGA‐325, TCGA‐LGG, and TCGA‐GBM were chosen for validation. The detailed clinicopathological characteristics including different grades of glioma patients were summarized in Table S1. The raw data from CGGA or TCGA were displayed in the form of fragments per kilobase of transcript per million fragments mapped (FPKM) and transformed to transcripts per kilobase million (TPM), while the raw CEL files in the REMBRANDT dataset were converted to expression matrix based on a GC‐Robust Microarray Averaging algorithm (GCRMA) algorithm for background adjustment and quantile normalization. The number of patients with survival data and gene expression values in the five datasets were listed in Table S2.

### 2.2 Copy number variation (CNV) calculation

Copy number variation information from TCGA datasets were obtained by TCGAbiolinks and mapped to GENCODE annotation (https://www.gencodegenes.org/) version 22 by bedtools software on Windows Subsystem Linux 2 (WSL2). Genes with focal CNV values less than −0.3 were regarded as “loss” (−1), while the CNV values greater than 0.3 were deemed as “gain” (+1) and values between and including −0.3–0.3 were defined as “neutral (0)”.^22^

### 2.3 Unsupervised clustering of expression profile of 59 ferroptosis‐associated genes (FRGs)

Since ferroptosis process is a consequence of interrupted dysfunction in oxidant metabolism, iron metabolism, lipid metabolism, energy metabolism, and other unclassified factors,^11^, ^23^, ^24^, ^25^ 60 FRGs which we selected belong to the five categories as previously described.^26^ However, there is a low expression level for *NOX1* in all the samples from the REMBRANDT dataset. Thus, we chose 59 FRGs for subsequent analysis. The classification of these FRGs was summarized in Table S3. Expression set of FRGs from the REMBRANDT dataset was subject to unsupervised cluster analysis using the Consensu Cluster Plus R package via k‐means clustering algorithm with Euclidean distance.^27^, ^28^

### 2.4 Analysis of differentially expressed genes (DEGs) and functional annotation

An empirical Bayesian method was employed for DEGs analysis by using limma R package.^29^ An adjusted P value less than 0.05 and absolute log2 fold change (log2FC) greater than 2 were considered as DEGs and employed for further functional annotation on gene ontology (GO) by clusterProfiler R package.

### 2.5 Construction of immune checkpoint blockage signatures and other functional signatures

Gene sets which could predict the responses to immune checkpoint blockade (ICB) therapy were obtained from the work by Mariathasan and his colleagues.^30^ Hallmark (h.all.v7.1.symbols), Kyoto Encyclopedia of Genes and Genomes (KEGG, c2.cp.kegg.v7.1.symbols), and GO (c5.all.v7.1.symbols) gene sets were downloaded from Molecular Signatures Database (www.gsea‐msigdb.org) and analyzed using GSVA R package.^31^

### 2.6 Depiction of tumor immune microenvironment (TIME) in glioma

The characteristics of TIME include infiltration of immune cells, activation of anti‐cancer immunity cycle and expression of immune checkpoints. In this study, we collected 667 immunomodulators including immune cells (Table S4) from the study of Charoentong research group^32^ using ssGSEA algorithm. As previously described, there were seven steps involving in the activation of anti‐cancer immunity cycle, which included release of cancer cell antigens (Step 1), cancer antigen presentation (Step 2), priming and activation (Step 3), trafficking of immune cells to tumors (Step 4), infiltration of immune cells into tumors (Step 5), recognition of cancer cells by T cells (Step 6) and killing of cancer cells (Step 7)^33^ and these steps could be scored by ssGSEA based on gene expression of each sample.^34^ The score of each step reflected the activation of anti‐tumor immunity. We then collected four immune checkpoints including PD‐L1, PD‐1, CTLA‐4, and IDO‐1 from Xu research group^35^ and they were regarded as key targets for glioma immunotherapy.

### 2.7 Construction of the risk score based on FRGs by random survival forest (RSF)

RSF model is an ensemble‐tree method that adapts the random forests to right‐censored data and survival analysis.^36^, ^37^ The REMBRANDT dataset was selected as training cohort and other three datasets including CGGA‐693, CGGA‐325, and TCGA cohorts were used for validation. Prior to the establishment and validation of RSF model, the expression profile of 59 FRGs in each sample were standardized. The selected FRGs based risk score by RSF was generated by the rfsrc function implemented in the randomForestSRC R package (kogalur.github.io/randomForestSRC). Based on the median value of risk score, the glioma patients were divided into two groups: high risk and low risk. The patients’ survival analysis was conducted using Kaplan–Meier (KM) curves and log‐rank tests were employed for analyzing statistical differences between high‐risk score and low‐risk score groups. The accuracy of risk score was evaluated using the receiver operating characteristic (ROC) curves.

### 2.8 Statistical analysis and Visualization

The expression profile of 59 FRGs was analyzed via Principal Component Analysis (PCA) with FactoMine R package. Correlations between variables were assessed via Spearman coefficient. Kruskal–Wallis tests were applied for the comparison of gene expression in two or more than two groups. The landscape of CNV and gene location were visualized by RCircos R package.^38^ The overall survival (OS) of the glioma patients between different groups was analyzed using Kaplan‐Meier curves with the log‐rank test. Univariate and multivariate Cox regression model were employed for calculating hazard ratios (HRs) and the coefficients of those regression models were visualized by Nomogram model. All statistical data were analyzed using R software (version 3.6.3). A *p* value less than 0.05 was considered as the statistical significance.

## 3 RESULTS

**FIGURE 1:**
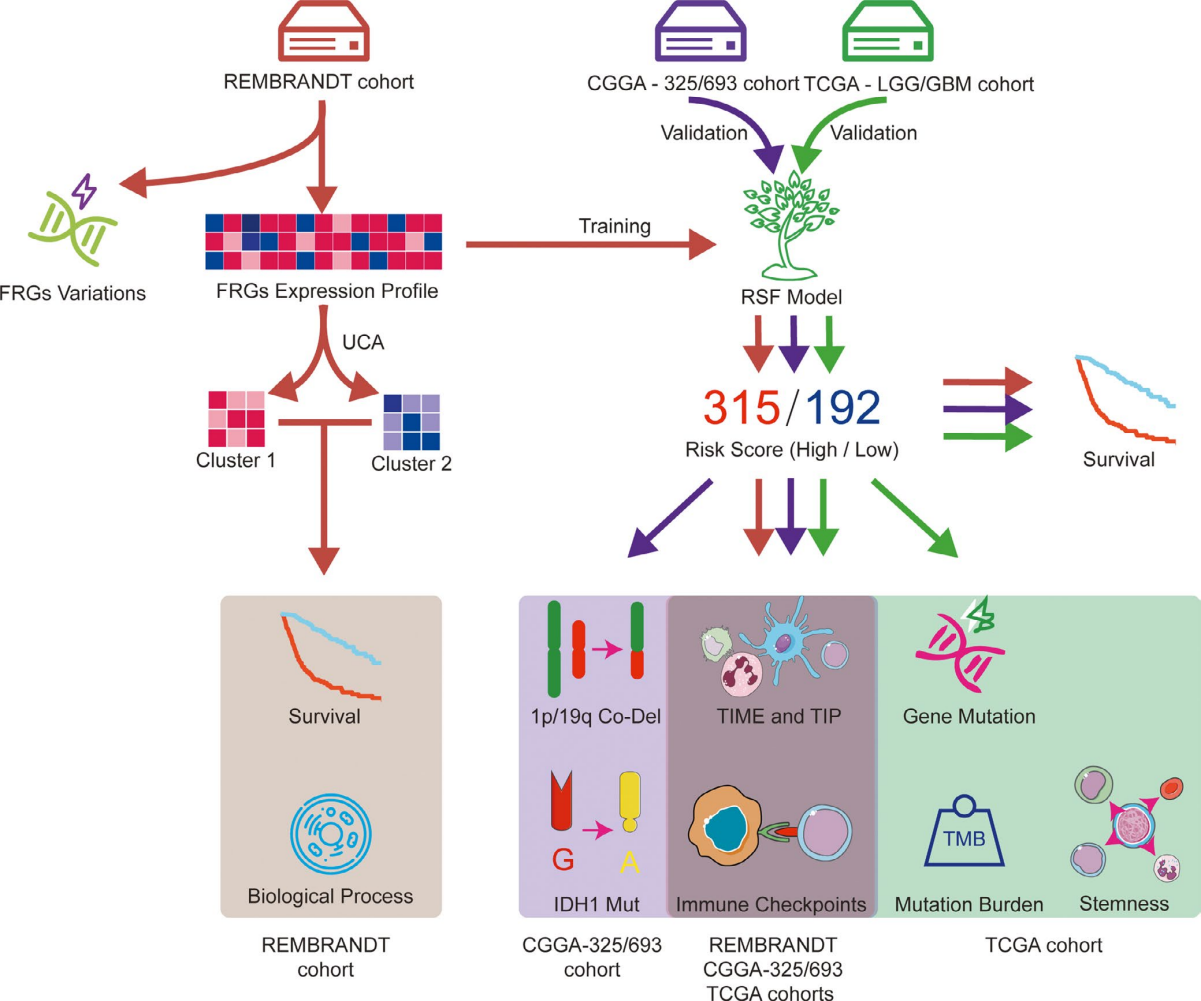
Workflow of data analysis in our present work. FRGs, ferroptosis‐related genes; RSF, random survival forest; TIME, tumor immune microenvironment; TIP, tracking tumor immunophenotype; TMB, tumor mutational burden; UCA, unsupervised clustering analysis

Figure 1 summarized the flow chart of data analysis. In the training stage, 405 glioma patients from the GSE108474‐REMBRANDT dataset were enrolled while four other datasets including TCGA‐GBM (151 patients), TCGA‐LGG (450 patients), CGGA‐693 (516 patients), and CGGA‐325 (291 patients) were employed for analysis in the validation stage. The detailed information of all these patients was listed in Table S1.

### 3.1 Landscape of FRGs in REMBRANDT and TCGA cohorts

**FIGURE 2:**
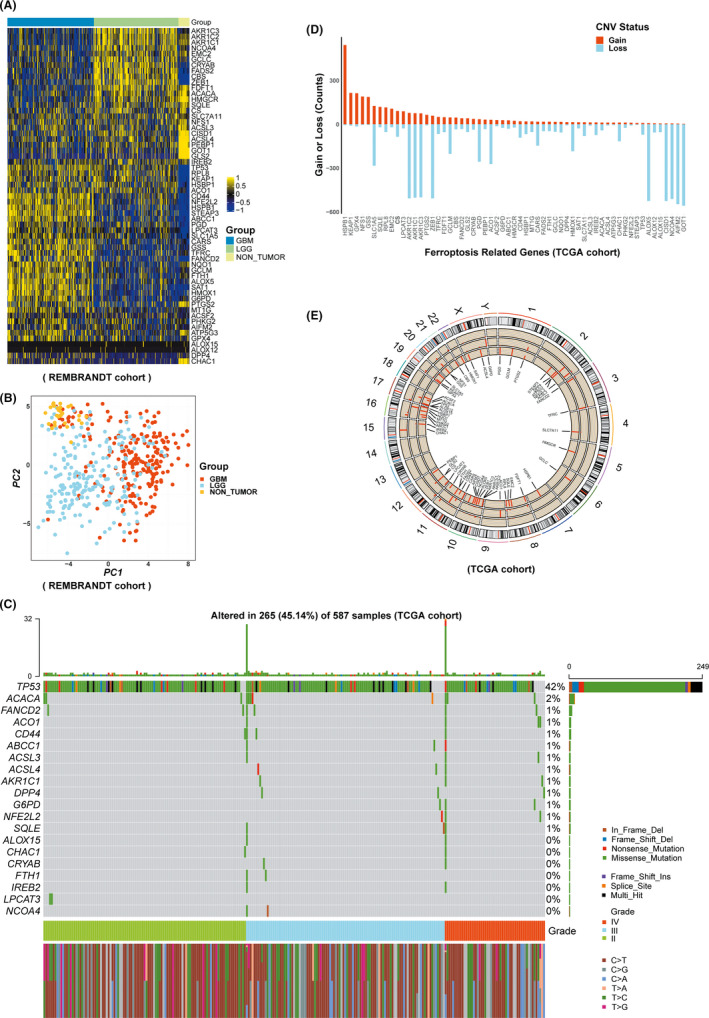
Landscape of ferroptosis‐related genes (FRGs) in REMBRANDT and TCGA cohorts. A, The differential expression of FRGs in GBM, LGG, and NON‐TUMOR tissue samples in REMBRANDT cohort. B, Indicates the principal component analysis results can be completely distinguished from GBM, LGG, and NON‐TUMOR groups on the basis of the expression of FRGs in REMBRANDT cohort. C, The variation of FRGs in TCGA cohort. D, CNV status analysis in TCGA cohort. E, The location and mutation frequency of CNV of 59 FRGs in TCGA cohort

The FRGs were conducted from two aspects including gene expression (in REMBRANDT cohort) and variation (in TCGA cohort). On the one hand, it was obvious that the majority of selected FRGs were differentially expressed among GBM, LGG, and NON‐TUMOR groups, except ALOX12 and ALOX15 (Figure 2A). Similarly, PCA analysis also demonstrated that the patients could be completely distinguished from three groups on the basis of the expression of FRGs (Figure 2B). On the other hand, the variation of FRGs was also evaluated in TCGA cohorts. It was found that there was the percentage of approximately 45.14% (265/587) glioma patients who displayed top 20 mutations of FRGs, with *TP53* having the highest mutation frequency (42%) and 19 other FRGs with the mutation frequency range from 0% to 2% (Figure 2C). In the meantime, CNV status analysis showed a frequent alteration in 59 FRGs. It was noted that *HSPB1* had the most significant copy number amplification while nine FRGs including *AKR1C2*, *AKR1C1*, *AKR1C3*, *ZEB1*, *ALOX5*, *CISD1*, *NCOA4*, *AIFM2*, and *GOT1* possessed the most widespread CNV deletion (Figure 2D). The location and mutation frequency of CNV of 59 FRGs were indicated in Figure 2E. Collectively, these results suggest distinct expression and variation of FRGs occurred in different grades of glioma.

### 3.2 Development of the FRGs‐related risk score in REMBRANDT cohort

**FIGURE 3:**
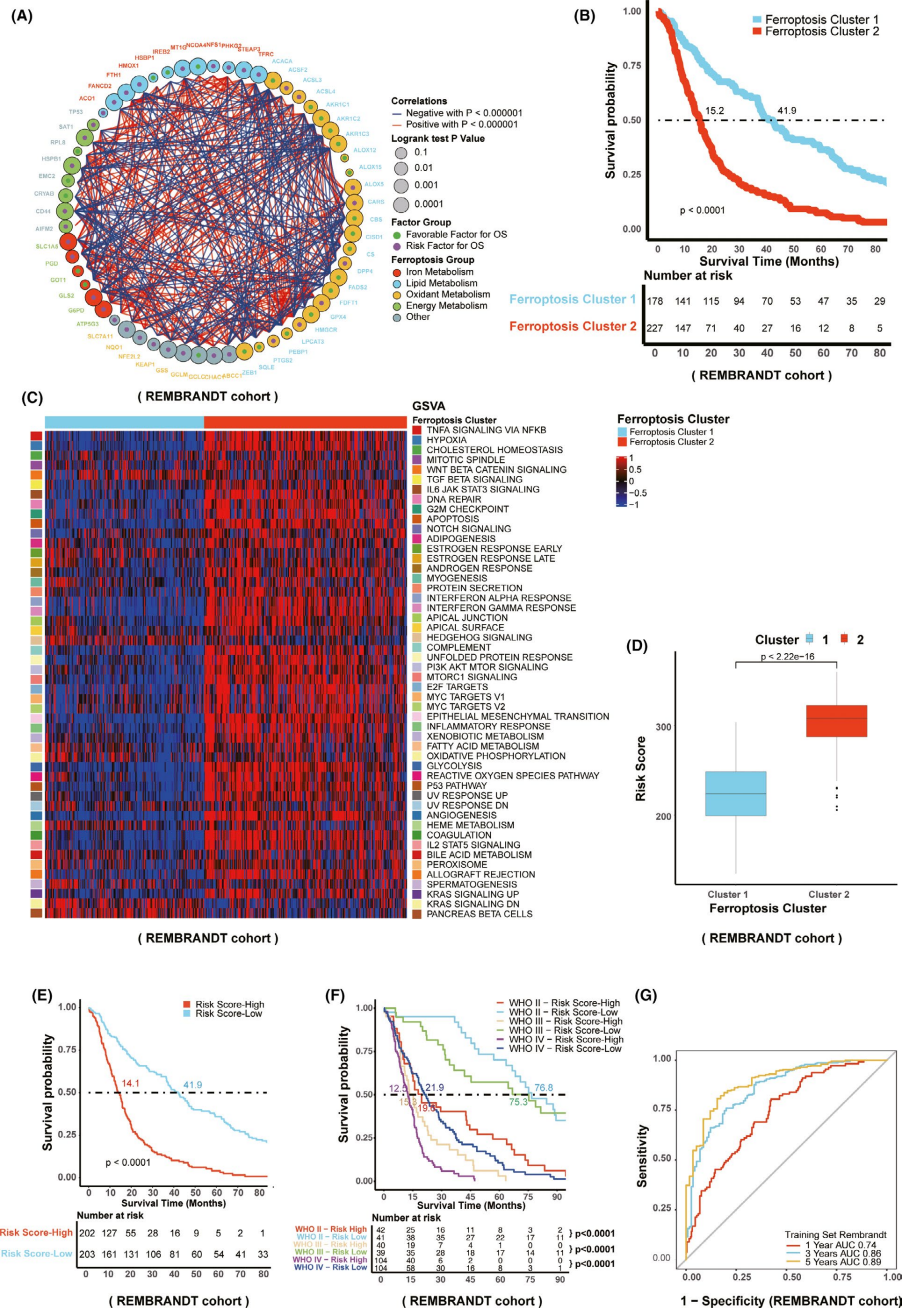
Development of the FRGs‐related risk score in REMBRANDT cohort. A, Shows 59 FRGs are divided into five categories including iron metabolism, lipid metabolism, oxidant metabolism, energy metabolism, and others. B, The relationship between distinct ferroptosis clusters and glioma patients’ prognosis. C, Indicates ferroptosis cluster 2 is associated with 50 well‐defined hallmark gene sets which are involved in all kinds of biological processes. D, The relationship between risk score and ferroptosis clusters. E, The relationship between risk score and clinical outcome of glioma patients. F, Effects of distinct risk score in different glioma grades according to WHO on the survival probability in glioma patients. G, Shows ROC curve analysis reflecting the predictive value of risk score in the glioma patients’ outcome in the aspect of 1 year AUC, 3 years AUC, and 5 years AUC

Next, we explored whether the correlation of FRGs and prognosis in glioma patients. As shown in Figure 3A, FRGs are generally divided into five categories including iron metabolism, lipid metabolism, oxidant metabolism, energy metabolism, and others, among which these FRGs were interacted with each other (Figure 3A and Figure S1A). Of note, most of genes associated with ferroptosis process in oxidant metabolism are risk factors for overall survival except GCLC. The detailed information showing hazard ratios of FRGs were summarized in Figure S1B. Furthermore, unsupervised cluster analysis was performed based on differential FRGs. We attempted to conduct cluster analysis range from two to five and it was found that two sorts of ferroptosis clusters including ferroptosis cluster 1 and ferroptosis cluster 2 were the most suitable to distinguish the glioma patients (Figure S2A–E). In the meantime, the patients in the ferroptosis cluster 1 exhibited better prognosis than those in ferroptosis cluster 2 group with the median survival time of 41.9 months and 15.2 months, respectively (Figure 3B). Besides, the poor prognosis in the patients who belong to the ferroptosis cluster 2 group displayed the majority of activated 50 hallmark gene sets which represents well‐known biological processes (Figure 3C and Table S5), reflecting the value of this cluster analysis in glioma outcome. In order to perform personalized assessment of the role of FRGs in glioma patients, RSF was constructed using the index risk score in our present work. As shown in Figure 3D, the patients in the ferroptosis cluster 1 had a lower risk score than those in ferroptosis cluster 2. The result of multivariate Cox regression analysis indicated that risk score was an independent predictive factor, which was similar to glioma grade (Figure S3A). Accordingly, a more satisfactory clinical outcome was observed in the low‐risk score group with the median survival time of 41.9 months (Figure 3E). Furthermore, in different grades of glioma patients, the high‐risk score group had poorer OS with the medial survival time of 75.3, 15.3, and 12.5 months in grade II, III, and IV, respectively (Figure 3F). ROC curve showed that the development of risk score in our present study exhibited a good predictive value in the aspect of 1 year AUC, 3 years AUC and 5 years AUC which were 0.74, 0.86, and 0.89, respectively (Figure 3G).

### 3.3 Validation of prognostic value of FRGs‐related risk score in CGGA‐693, CGGA‐325, and TCGA cohorts

**FIGURE 4:**
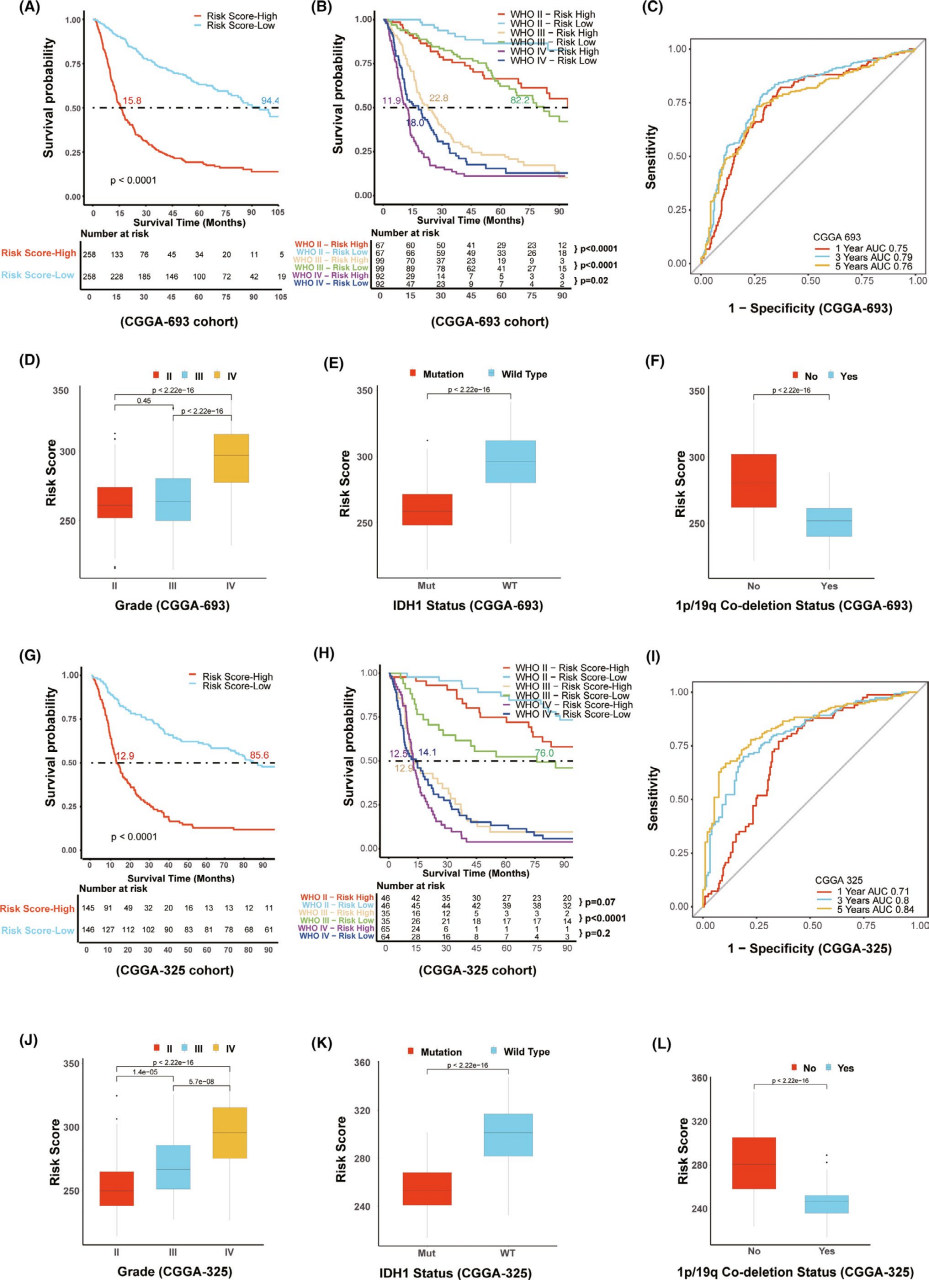
Validation of prognostic value of risk scores in CGGA‐693 and CGGA‐325 cohorts. A and G, The relationship between risk score and glioma patients’ prognosis in CGGA‐693 and CGGA‐325, respectively. B and H, Effects of distinct risk score in different glioma grades according to WHO on the patients’ survival. C and I, Validation of the predictive value of risk score in the aspect of 1 year AUC, 3 years AUC and 5 years AUC via ROC curve analysis in CGGA‐693 and CGGA‐325 cohorts, respectively. D and J, Correlations between risk score and glioma grades in CGGA‐693 and CGGA‐325, respectively. E and K, Correlation between risk score and IDH mutation in CGGA‐693 and CGGA‐325, respectively. F and L, Correlation between risk score and 1p/19q co‐deletion in CGGA‐693 and CGGA‐325, respectively

We further validated the prognostic value in various datasets including CGGA‐693, CGGA‐325, and TCGA via distinct risk score groups. Consistent with the results in the REMBRANDT dataset as shown above, the risk score was also validated to serve as an independent predictive factor by multivariate Cox regression analysis in the three cohorts (Figure S3B–D). Besides, it was confirmed that the glioma patients in the high‐risk score group of CGGA‐693, CGGA‐325, and TCGA cohorts displayed poor prognosis with the median survival time of only 15.8 months, 12.9 months, and 19.3 months, respectively (Figure 4A, Figure 4G, and Figure S4A). In different grades of glioma patients (including grade II, III, and IV), the high‐risk score group displayed shorter OS than that in the low‐risk score group (Figure 4B, Figure 4H, and Figure S4B) although different risk scores in grade II or grade IV of CGGA325 dataset and grade IV in TCGA cohort had a minimal effect on patients’ survival. ROC curve also validated a good predictive value in the survival time of 1 year, 3 years, or 5 years (Figure 4C, Figure 4I, and Figure S4C). In addition, we also analyzed the role of risk score in the aspect of pathological grade, IDH status, and 1p/19q co‐deletion state in CGGA‐693 and CGGA‐325 cohorts, which indirectly reflect the outcome of glioma patients.^39^ It was interesting that glioma patients with grade IV, IDH wild type and no deletion of 1p/19q had the high‐risk score (Figure 4D–F and Figure 4J–L). In these two datasets (CGGA‐693 and CGGA‐325), Nomogram model was also established via integration of risk score, grade, IDH status, and 1p/19q co‐deletion state to assess the survival prediction in glioma patients (Figure S5A). It was found that this model could well predict patients’ survival in the aspects of 1 year OS, 3 year OS, and 5 year OS in both CGGA‐693 and CGGA‐325 cohorts (Figure S5B,C). Compared with analysis based on the WHO grade (AUC at 0.77, 0.78, and 0.78, respectively, in terms of 1 year OS, 3 year OS, and 5 year OS), our established Nomogram model which involved risk score had better predictive value with AUC at 0.81, 0.84, and 0.83, respectively, in CGGA‐693 dataset (Figure S6A–C). Consistent results were also obtained in CGGA‐325 dataset although there was no significant difference between WHO grade and Nomogram groups in the aspect of 1 year OS (Figure S6D–F). Altogether, these data verify the important prognostic value of distinct risk scores in glioma patients.

### 3.4 Evaluation of immunotherapy in glioma in REMBRANDT cohort with distinct risk scores

**FIGURE 5:**
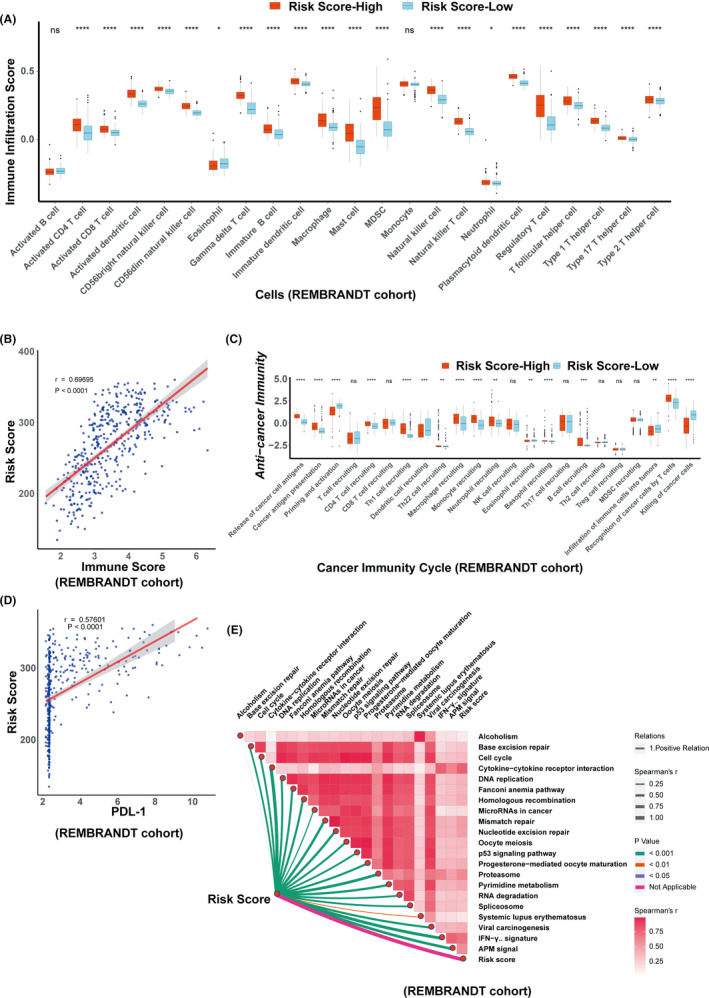
Evaluation of immunotherapy in glioma with risk score in REMBRANDT cohort. A, The relationship between risk score and immune infiltration score. B, Shows the relationship of the risk score and the immune score. C, Correlation between the risk score and cancer immunity cycles. D, Correlation between the risk score and PD‐L1 expression. E, Shows spearman correlation analysis reflecting the relationship of risk score and immune checkpoint blockade‐related pathways

Since prior work has illustrated that ferroptosis process is involved in tumor immunotherapy [11], we would like to explore whether ferroptosis is correlated with glioma immunity via bioinformatics. Analysis of immune cell infiltration demonstrated the abundance of innate immune cell infiltration including natural killer cell, macrophage, mast cell, MDSC, plasmacytoid dendritic cell except eosinophil in the high‐risk score group (Figure 5A). Accordingly, immune score defined by 23 categories of immune cells using the ssGSEA algorithm was also positively correlated with the high‐risk score group via spearman correlation analysis (Figure 5B). The cancer immunity cycle is a critical index for evaluating the biological functions of the chemokine system and other immunomodulators.^33^, ^34^ In the high‐risk score group, activities of various steps in the cycle were observed to be upregulated including the release of cancer cell antigens (Step 1), cancer antigen presentation (Step 2), immune cells recruiting (Step 4) (CD4 T cell recruiting, Th1 cell recruiting, Th22 cell recruiting, Macrophage recruiting, Monocyte recruiting, Neutrophil recruiting, NK cell recruiting, Basophil recruiting, and B cell recruiting) and recognition of cancer cells by T cells (Step 6) (Figure 5C). The elevated activities of these steps predicted the potent immunological potential. To our surprise, our results showed that the killing of cancer cells (Step 7) was weaker in the high‐risk score group than that in the low‐risk score group. It may be due to the high expression of PD‐L1, as shown in Figure 5D. Besides, we also made an analysis of the relationship between distinct risk score and ICB‐related pathways. It was found that the majority of the patients in the high‐risk score group were positively correlated with the enrichment scores for ICB‐related positive signatures, except systemic lupus erythematosus (Figure 5E and Table S6). Collectively, these data suggest that the development of risk score associated with FRGs can well predict the glioma immunotherapy.

### 3.5 Validation of the immunotherapy in glioma in CGGA‐693, CGGA‐325, and TCGA cohorts with distinct risk scores

**FIGURE 6:**
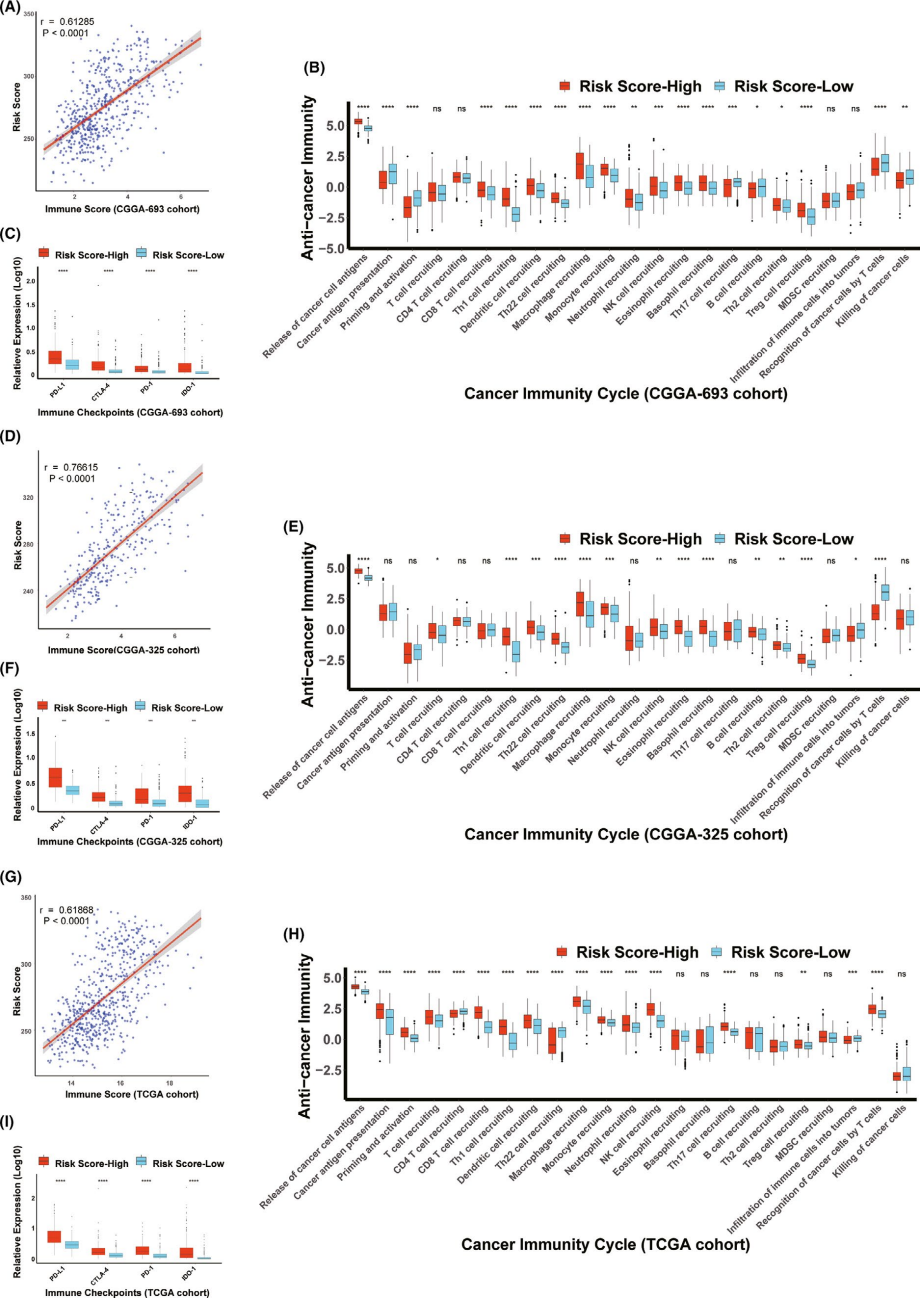
Validation of the immunotherapy in glioma with risk scores in CGGA‐693 and CGGA‐325 and TCGA cohorts. A,D and G, Validation of correlation between the risk score and the immune score. B,E and H Verification of correlation between the risk score and cancer immunity cycles. C,F and I Validation of correlations between the risk score and immune checkpoints

The validation of the immunotherapy in glioma was conducted via analysis of diverse cohorts including CGGA‐693, CGGA‐325, and TCGA. Immune score was confirmed to be positively correlated with risk score in these cohorts (Figure 6A, Figure 6D, and Figure 6G). With respect to the cancer immunity cycle, upregulations of the release of cancer cell antigens (Step 1) and immune cells recruiting (Step 4) (Th1 cell recruiting, Macrophage recruiting, Monocyte recruiting, and NK cell recruiting) were ascertained to be found in the high‐risk score group (Figure 6B, Figure 6E, and Figure 6H). Besides, the patients with high‐risk score exhibited the high expression of PD‐L1 in CGGA‐693, CGGA‐325, and TCGA cohorts, which was consistent with the results found in training dataset (REMBRANDT) (Figure 6C, Figure 6F, and Figure 6I). Intriguingly, other immunological indices including PD‐1, CTLA4, and IDO‐1, which are closely related to glioma progression,^35^ were also found to be abundantly expressed in the high‐risk score group (Figure 6C, Figure 6F, and Figure 6I). Consistent with the results shown in REMBRANDT cohort, the patients in the high‐risk score group in other datasets including CGGA‐693, CGGA‐325, and TCGA cohorts were positively associated with the enrichment scores for ICB‐related positive signatures, except RNA degradation (CGGA‐693, CGGA‐325, and TCGA cohorts) (Figure S7A–C, Table [Link], [Link], [Link]). Additionally, in TCGA cohort, tumor mutational burden (TMB), immunogenic mutation, DNA methylation stemness indices (mDNAsi), and RNA methylation stemness indices (mRNAsi), which are proved to be associated with ICB therapy,^40^, ^41^ were also analyzed in our present work. It was noted that elevations of TMB, immunogenic mutation and mDNAsi were observed in the high‐risk score group while there was a reduction of mRNAsi in glioma patients with high‐risk score (Figure S8A‐D). Taken together, these results again confirm the critical role of glioma immunotherapy via risk score.

## 4 DISCUSSION

Our present work systematically analyzed the landscape of 59 FRGs in glioma tissues and their associations with patients’ prognosis. More importantly, RSF model was constructed using risk score based on ferroptosis signature in glioma patients in REMBRANDT cohort and it was validated in other datasets including CGGA‐693, CGGA‐325, and TCGA cohorts. The glioma patients with low‐risk score exhibited a more satisfactory clinical outcome than those patients with high‐risk score. Additionally, immune signaling was also activated in the high‐risk score group, which indicates that the glioma patients with high‐risk score may have good response to immunotherapy.

Selective induction of cell death functions as a promising strategy in anti‐cancer research. In recent years, it is intriguing that induction of ferroptosis, a recently discovered RCD by Stockwell research group, has been shown to block tumorigenesis and metastatic process.^42^, ^43^ With respect to glioma research, the theme on the role of ferroptosis process in tumor progression particularly attracts considerable attention. Three classical ferroptosis‐related categories including system X_c_^−^ inhibition (xCT), *GSH* depletion, and *GPX4* blockade have been reported to serve as contributing factors of gliomagenesis.^44^, ^45^ Inhibition of xCT and depletion of *GSH* by pseudolaric acid B have been demonstrated to exert anti‐cancer effect on glioma.^44^ In the meantime, decrease of *GPX4* expression by the natural compound withaferin A was also previously found to trigger ferroptotic death in neuroblastoma cells.^46^ However, the comprehensive evaluation of the relationship between gene signature involving in ferroptosis process and glioma prognosis and therapeutic efficacy is still uncompleted. In our present work, we revealed that most of selected FRGs was differentially expressed in patients with different pathological grades and NON‐TUMOR control group. It was surprising that *ALOX12* and *ALOX15*, which are two members of lipoxygenase family, seemed to be unaltered due to very low expression in these groups. In consistent with our result, a previous study also illustrated unalterations of *ALOX12* and *ALOX15* in different glioma grades.^47^ In contrast, it was intriguing that the differential expression of *ALOX5* was observed among GBM, LGG, and NON‐TUMOR tissues. And this gene had the most widespread CNV deletion. These data altogether imply that one of lipoxygenase family members *ALOX5*, which belongs to FRGs, is critical for glioma progression. Furthermore, a signature from 59 FRGs could well predict the prognosis of glioma patients. In detail, the patients in the ferroptosis cluster 1 group exhibited satisfactory prognosis with the median survival time of 41.9 months. It indicates a good predictive value for glioma patients via ferroptosis gene signature.

Risk score of FRGs signature via RSF was also developed in our present work for the implementation of personalized evaluation of glioma patients. It was demonstrated that the high‐risk score group displayed poor prognosis in REMBRANDT cohort and this result was validated in three datasets including CGGA‐693, CGGA‐325, and TCGA. Further analysis illustrated that the patients with high‐risk score had worse pathological grades. In addition, other factors including IDH mutation status and 1p/19q co‐deletion state, which are recognized to be critical for glioma progression,^39^, ^48^ were also found to be associated with distinct levels of risk score. These results suggest that development of risk score is negatively correlated with patients’ prognosis.

Immunotherapy is widely applied in various cancers. Nowadays, there are diverse immunotherapeutic approaches available such as immune checkpoint inhibitors, peptide vaccines, dendritic cells vaccines, chimeric antigen receptor‐T cells, and oncolytic viruses for treating glioma.^49^ Also, there are some genes including coatomer protein complex subunit beta 2,^50^ transmembrane protein 71,^51^ and regulators of G protein singling 16^52^ which serve as prognostic factors via targeting glioma immunity. These data altogether implicate that altered immunoreactive response can influence glioma progression. In our present work, functional annotation of FRGs‐based risk score showed that immune signaling was highly associated with different levels of risk score in glioma. In detail, it was observed that abundant expressions of immune cell infiltration and immune checkpoints notable PD‐L1, PD‐1, CTLA‐4 and IDO‐1, and TMB as well as immunogenic mutation in the high‐risk score group. It indicates the association of ferroptosis signature and glioma immunity. In fact, there were several previous investigations supporting that intervention of ferroptosis process had a significant effect on cancer immunotherapy.^13^, ^53^ Deficiency of the FRG (SLC7A11) in mice with melanoma was more susceptible to anti‐PD‐L1 therapy,^54^ which suggests again the role of ferroptosis in anti‐tumor immunity. The evidence supporting the role of FRGs in tumor immunity also arises from the study that T cells lacking GPX4 failed to expand and they rapidly underwent ferroptotic cell death.^55^ GPX4‐mediated maintenance of redox homeostasis was vital for stimulator‐of‐interferon genes (STING) activation,^56^ which plays a critical role in initiating innate immune responses against tumors and microbial infection. In addition, the immune‐related indices shown above are also well‐recognized factors which involve tumor immunotherapy,^35^, ^40^, ^57^, ^58^ which also suggests that construction of risk score based on FRGs could well predict glioma immunotherapy.

Undoubtedly, there are also other considerations to be clarified. Firstly, our current results are obtained merely in public databases and it is indispensible to validate our bioinformatics analysis in experimental research. The clinical value of risk score in the prediction of prognosis and immunotherapy in glioma should also be confirmed in the future. Additionally, the detailed mechanism of each of FRGs in glioma progress is also explored in the coming years. In any case, our current work provides the theoretical base for the prediction of FRGs signature in glioma prognosis and immunotherapy. From the translational aspect in clinical settings, the FRGs‐based risk score which we established in the current work may have a predictive role in glioma immunotherapy.

## CONFLICT OF INTEREST

All the authors declared that there was no potential conflict of interest.

## AUTHOR CONTRIBUTIONS

XYM designed the study. RJW and WP analyzed the data and generated the figures. XYM involved in manuscript writing. QXX and HHZ revised the manuscript.

## Supporting information

Fig S1

Fig S2

Fig S3

Fig S4

Fig S5

Fig S6

Fig S7

Fig S8

Table S1

Table S2

Table S3

Table S4

Table S5

Table S6

Table S7

Table S8

Table S9

## Data Availability

GSE108474 dataset is available in the Gene‐Expression Omnibus at https://www.ncbi.nlm.nih.gov/geo, TCGA‐LGG and TCGA‐GBM datasets are available in the Cancer Genome Atlas at https://portal.gdc.cancer.gov/, and CGGA‐693 and CGGA‐325 datasets are available in the Chinese Glioma Genome Atlas at http://www.cgga.org.cn/.
