# Supplementary figures and images for: Ferroptosis‐related gene signature predicts prognosis and immunotherapy in glioma
Source: CNS Neurosci Ther. 2021 May 10;27(8):973–86. doi: 10.1111/cns.13654 (PMC8265949; doi:10.1111/cns.13654)

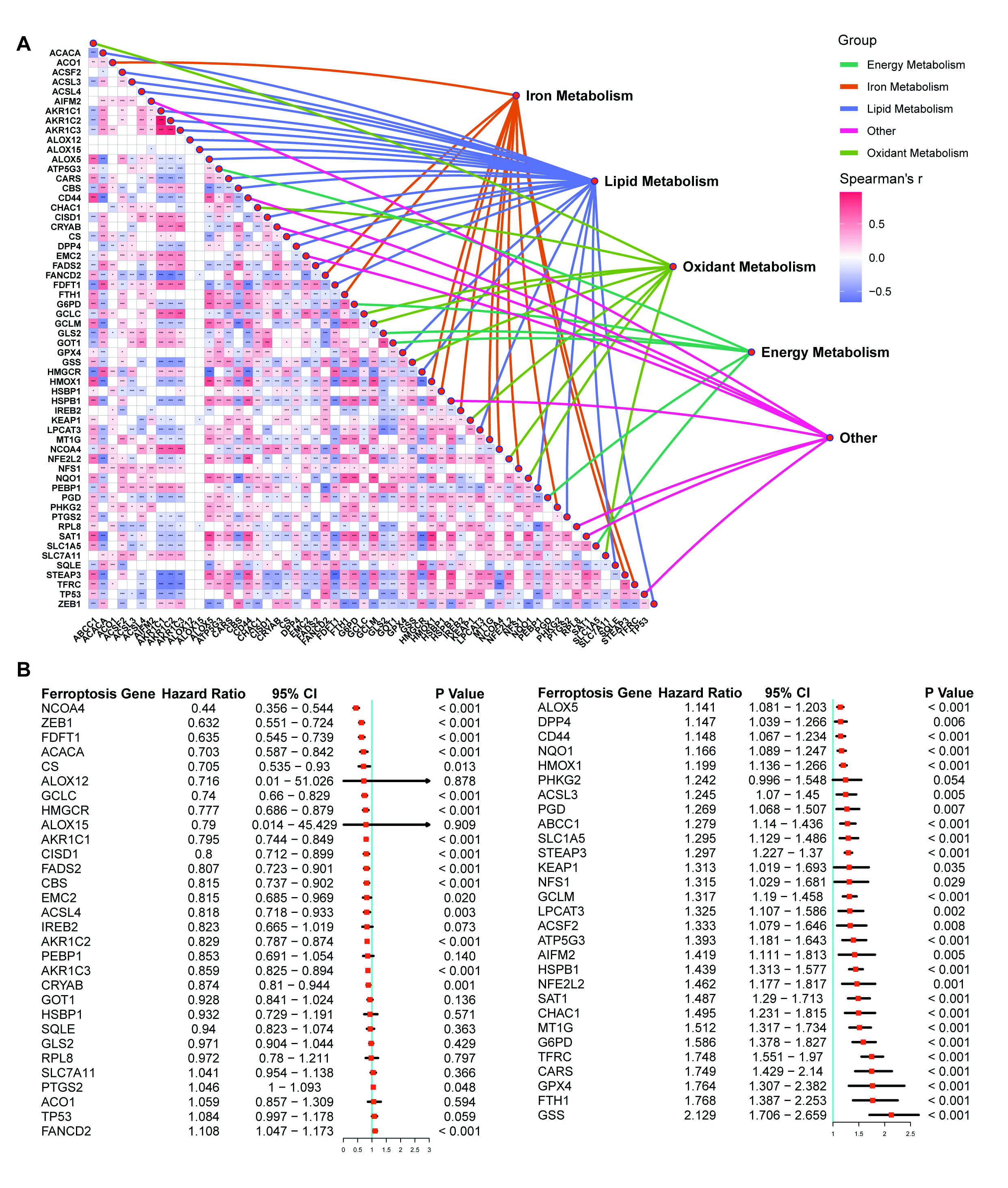

Supplement: Supplementary file 1 — Fig S1 [file CNS-27-973-s009.tif]

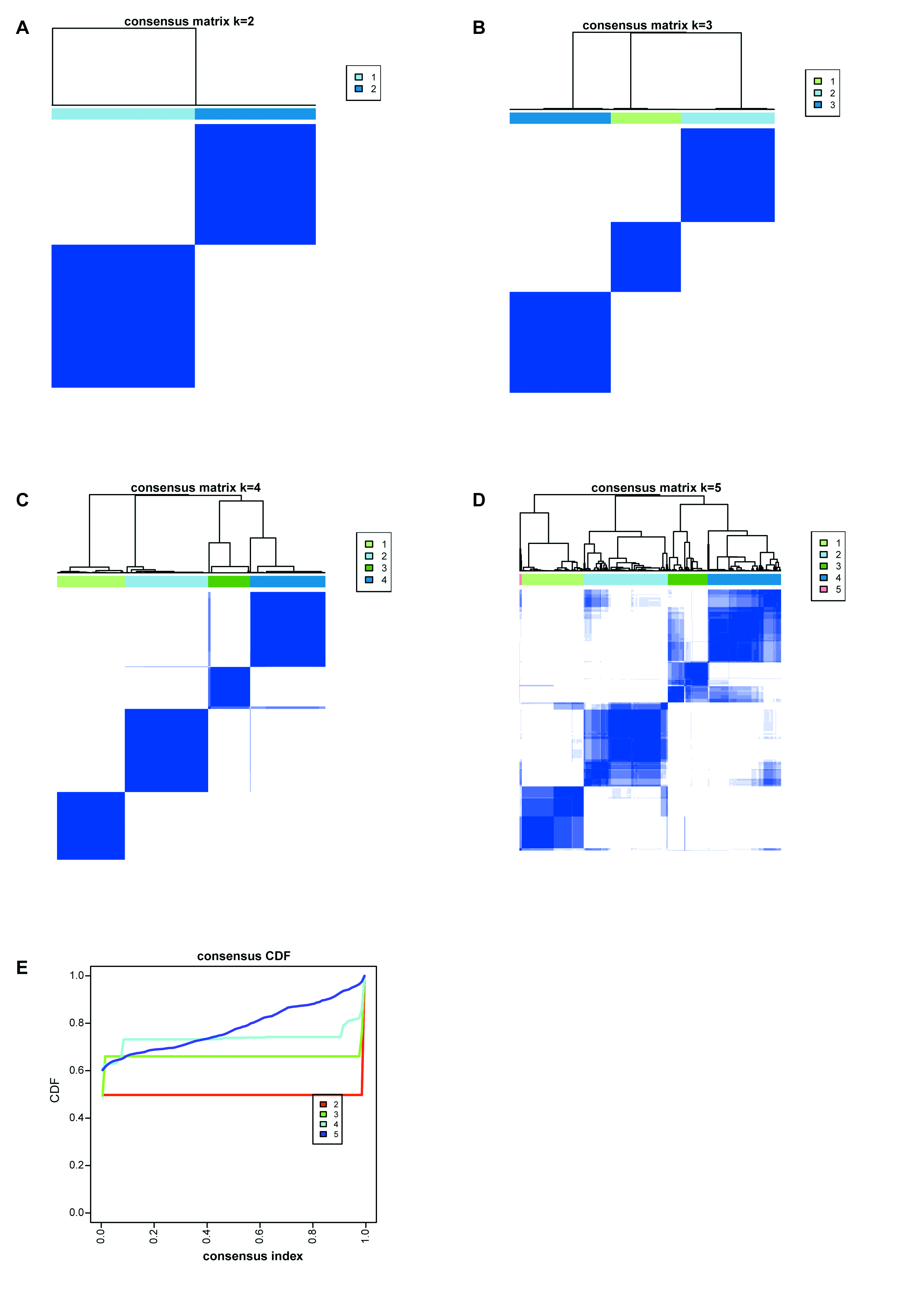

Supplement: Supplementary file 2 — Fig S2 [file CNS-27-973-s014.tif]

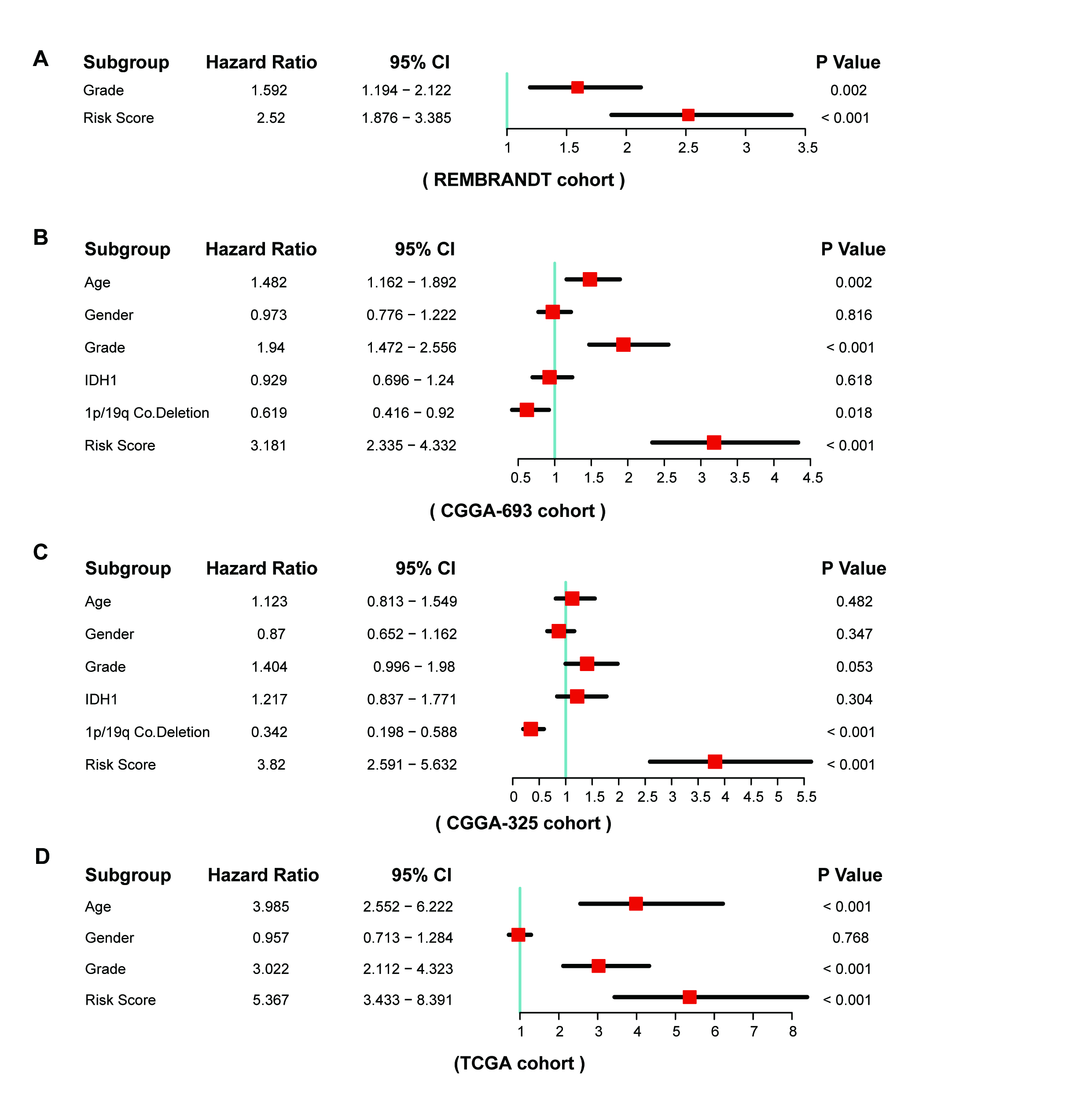

Supplement: Supplementary file 3 — Fig S3 [file CNS-27-973-s003.tif]

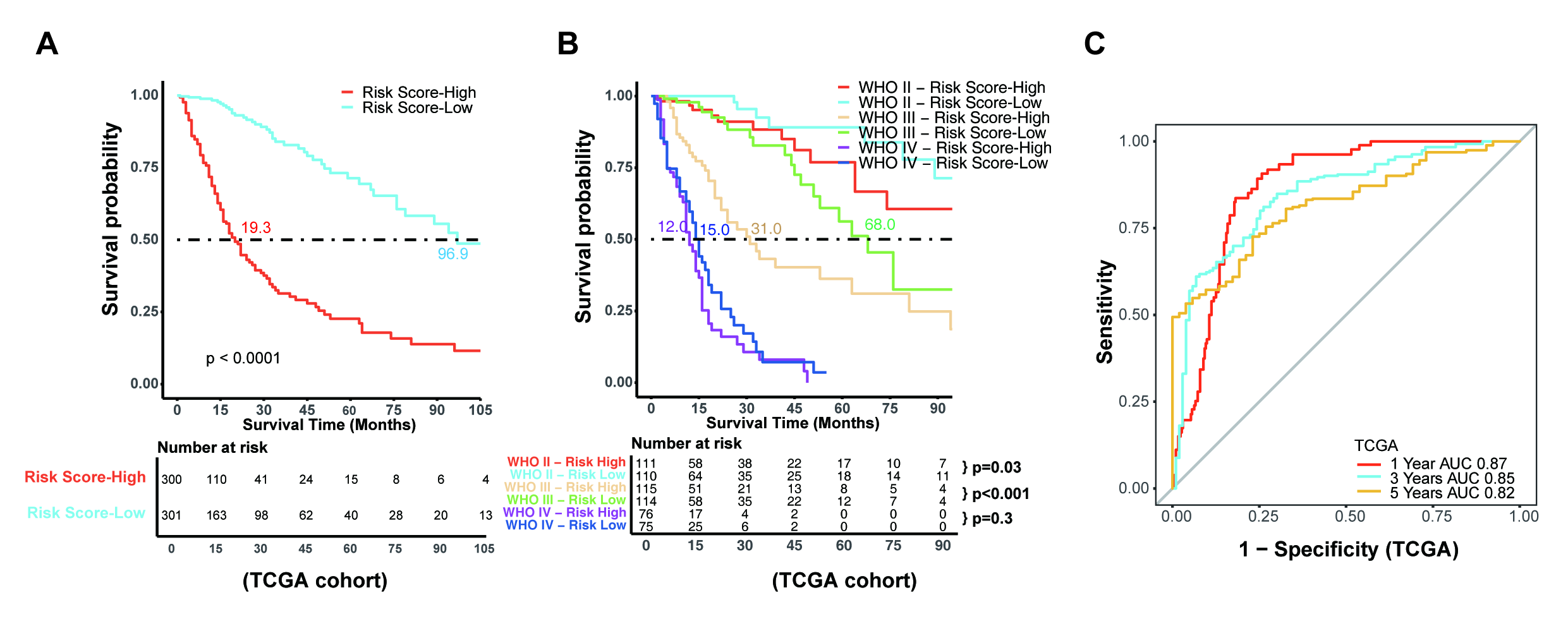

Supplement: Supplementary file 4 — Fig S4 [file CNS-27-973-s013.tif]

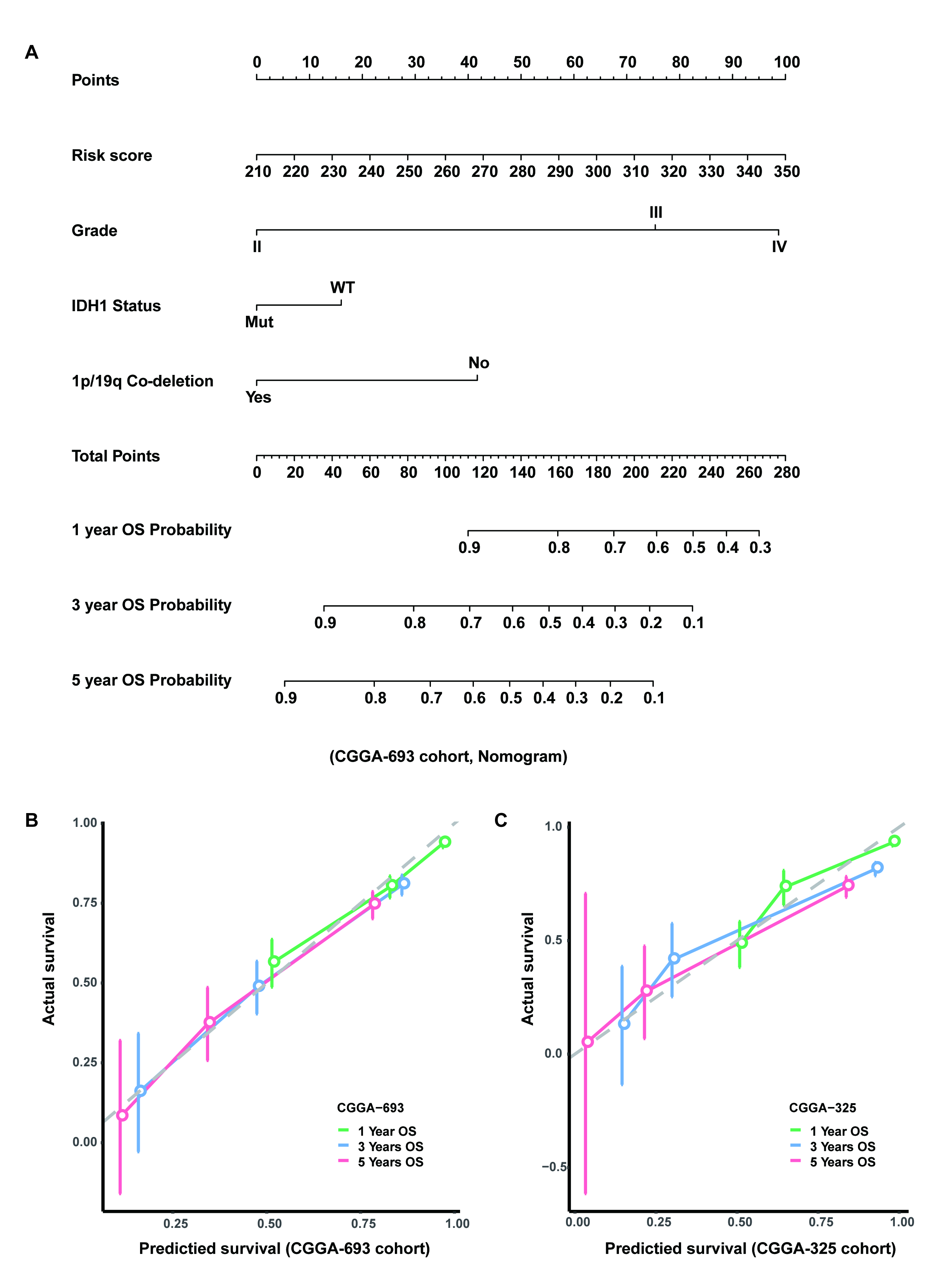

Supplement: Supplementary file 5 — Fig S5 [file CNS-27-973-s006.tif]

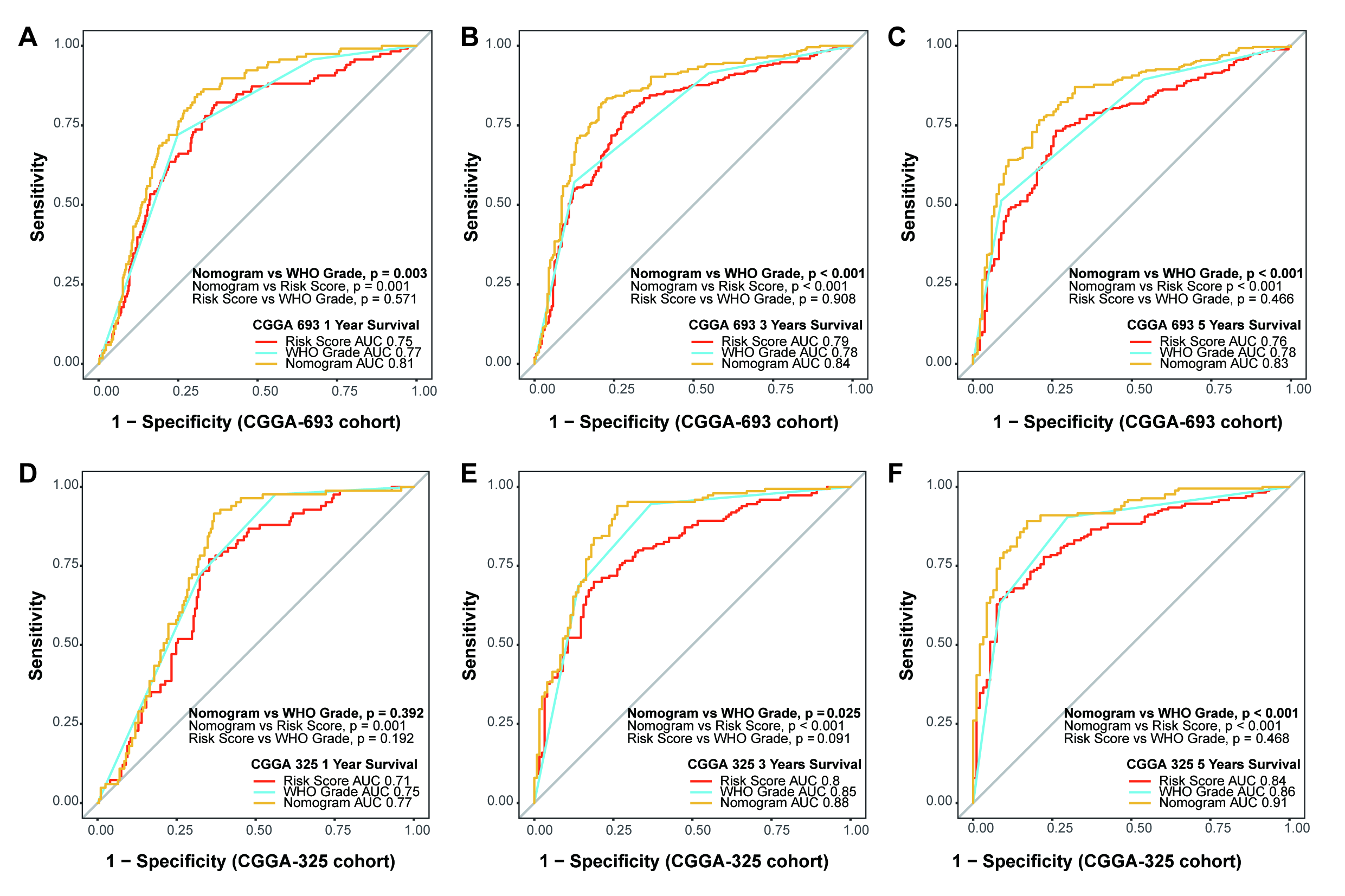

Supplement: Supplementary file 6 — Fig S6 [file CNS-27-973-s016.tif]

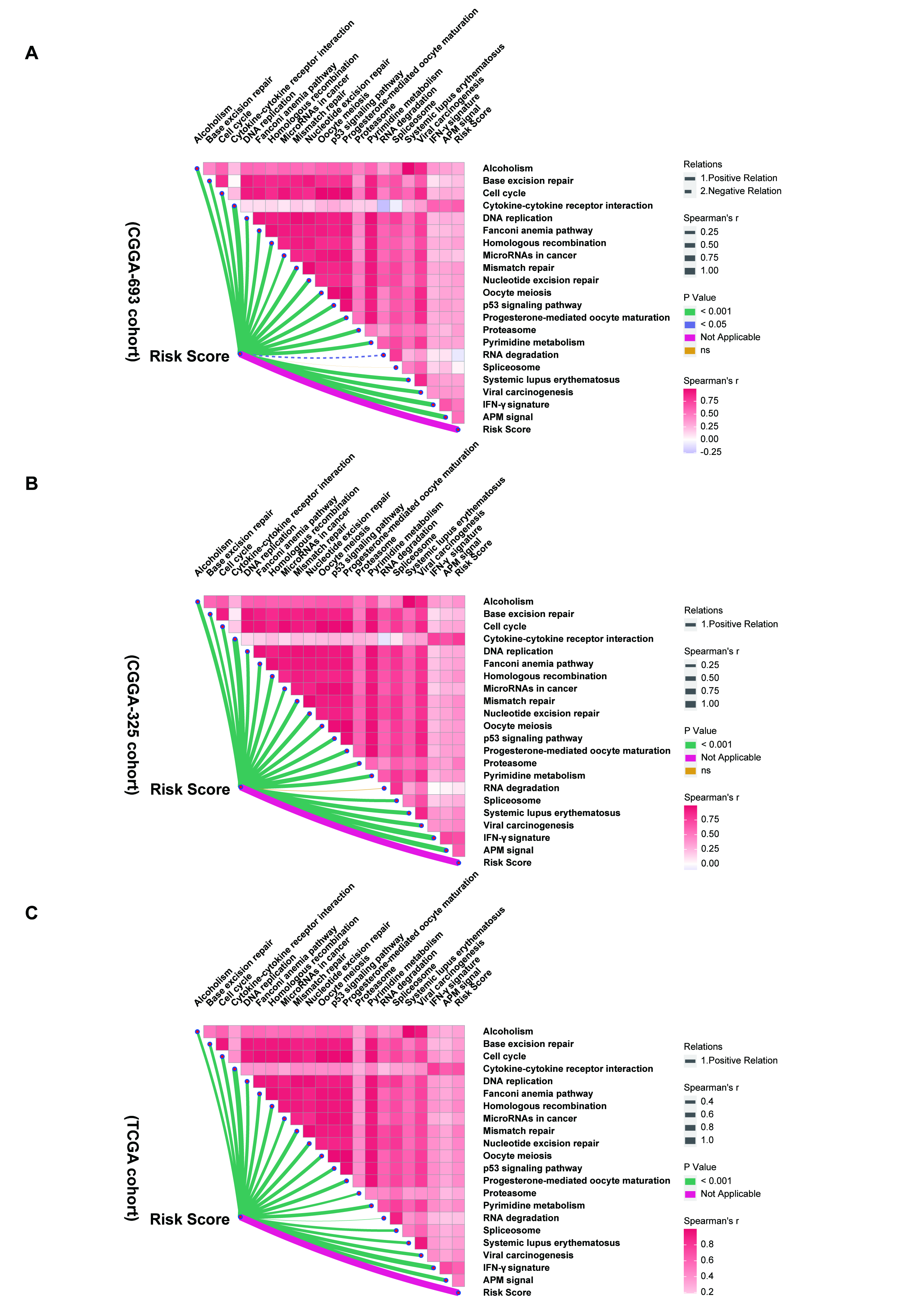

Supplement: Supplementary file 7 — Fig S7 [file CNS-27-973-s008.tif]

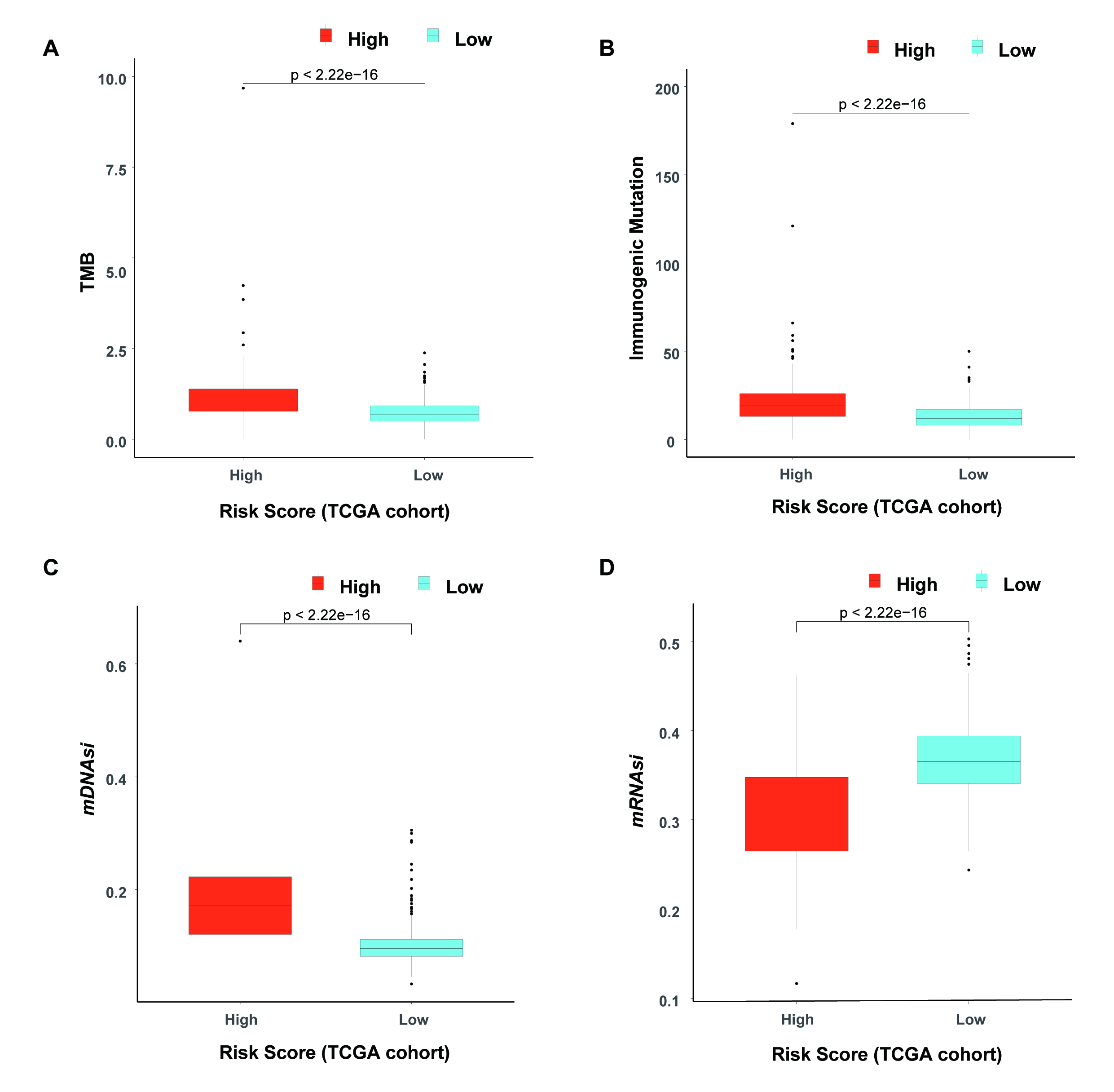

Supplement: Supplementary file 8 — Fig S8 [file CNS-27-973-s007.tif]
